# Hybrid Energy Harvesting Applications of ZnO Nanorods for Future Implantable and Wearable Devices

**DOI:** 10.3390/mi16060605

**Published:** 2025-05-22

**Authors:** Kathalingam Adaikalam, Hyun-Seok Kim

**Affiliations:** 1 Millimeter-Wave Innovation Technology Research Center, Dongguk University, Seoul 04620, Republic of Korea; kathu@dongguk.edu; 2 Division of Electronics and Electrical Engineering, Dongguk University, Seoul 04620, Republic of Korea

**Keywords:** solar energy conversion, oxide materials, dye-sensitized solar cell, ZnO nanorods, nanogenerator, piezogenerator, nanolevel energy convertor

## Abstract

The currently used electrical energy devices for portable applications are in limited life and need of frequent recharging, it is a big bottleneck for wireless and transportation systems. The scientific community is motivated to find innovative and efficient devices to convert environmental energy into useful forms. Nanogenerator can mitigate this issue by harvesting ambient energy of different forms into useful electrical energy. Particularly flexible nanogenerators can efficiently convert ambient mechanical energy into electrical energy which can be fruitfully used for self-powered sensors and electronic appliances. Zinc oxide is an interesting photosensitive and piezoelectric material that is expected to play a vital role in the synergetic harvesting of environmental thermal, sound, mechanical, and solar energies. As ZnO can be synthesized using easy methods and materials at low cost, the conversion efficiencies of solar and other energy forms can increase considerably. ZnO is a versatile material with interesting semiconducting, optical, and piezoelectric properties; it can be used advantageously to harvest more than one type of ambient energy. The coupled semiconducting and piezoelectric properties of ZnO are attractive for fabricating nanogenerators capable of harvesting both ambient optical and mechanical energies simultaneously. These nanolevel conversion devices are much required to power remote and implantable devices without the need for additional power sources. The present review briefly discusses the principles and mechanisms of different energy harvesting abilities of ZnO nanorods and their composites by consolidating available literature. In addition, the developments taking place in nanogenerators of different kinds—such as photovoltaic, piezoelectric, pyroelectric, and triboelectrics for self-powered technology—and their progress in hybrid energy harvesting application is reviewed.

## 1. Introduction

**Figure 1:**
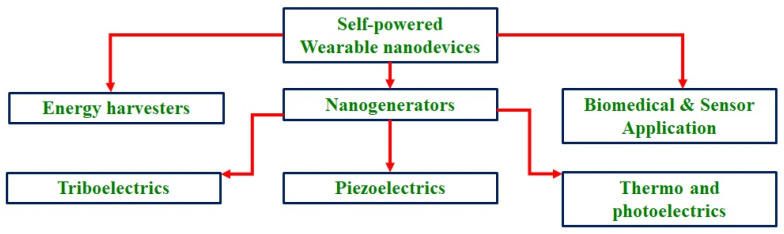
Self-powered wearable device applications and their different forms.

**Figure 2:**
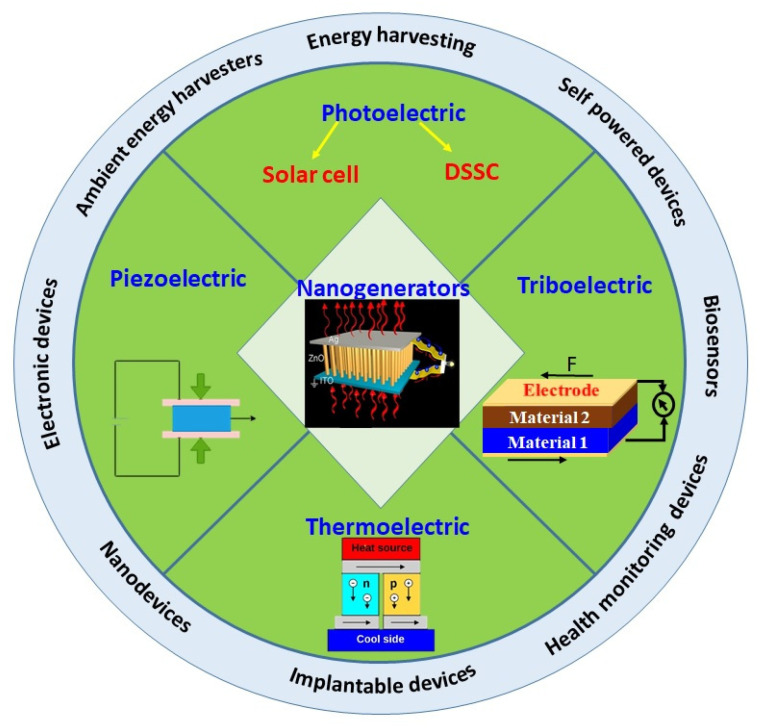
Schematic of different forms of nanogenerators and their applications.

The global energy demand is rapidly accelerating as a result of increased economic and industrial growth. Recently, there has been a fast change in industrialization, which largely exploits natural resources, leading to scarcity of energy and its related sources. It creates lots of environmental challenges in terms of pollution and resources depletion causing major challenges in the coming years. This situation forces us to find alternative sources of energy that support sustainability without degrading the environment [1]. These problems can be avoided by developing advanced materials and methods to utilize the environmental waste energies and renewable sources of energy. In the present scenario, conversion of solar light using photovoltaics (PVs) is expected to significantly impact global energy production in the future, considering the environmental hazardous effects of fossil fuels [2]. Solar energy is a clean and freely available renewable source of energy, which is largely used currently as pollution-free energy [3,4]. The solar cell’s function highly depends on the availability of sunlight, so it cannot be efficient all day. Instead, the triboelectric, piezoelectric, and thermoelectric effects combined with hybrid solar cells can be potential ambient energy harvesters suitable for a variety of applications. Similar to solar energy, there are other forms of renewable sources such as water, wind, and others. However, these sources and their current techniques cannot be used directly for miniatured and wearable devices, as these sources of energy are used for large scale production of energy, which again needs storage and transportation facilities. Compared to these sources, the sound, heat, and mechanical energies are freely available in the environment at all times [5]. Apart from heat, light, and sound, there are other forms of energies like chemical, biological, and human physical activities related energies. Among these various forms of energies, mechanical energy is the most ubiquitously available, derived from human activities and other developmental works [6]. These environmental energies can be easily converted using piezoelectric, pyroelectric, triboelectric, thermoelectric, and photovoltaic nanogenerators yielding low-cost and eco-friendly energy useful for various applications including wearable and implantable devices [7,8]. Wearable devices can convert human body heat and movements into energy, making them useful for powering wearable devices and contributing to a sustainable environment. These wearable devices have become essential for human life for monitoring health conditions and powering various portable communication devices [9]. The wearable piezoelectric devices will be game changing inventions in medical applications, they can be used as health monitoring devices. Recently, McDonald et al. has published a work on piezoelectric sensor to record heart function with high precision [10]. The wearable devices are self-powered nanogenerators, useful for energy harvesting from ambient environmental energies based on tribo, piezo, thermos, and photoelectric effects (Figure 1). The various principles of nanogenerators and their uses are also presented in Figure 2.

These miniatured nanolevel devices can make dramatic advances in this technological world to produce electronic devices having low power consumption, maintenance-free operation, and wireless communication with multilevel functions. Hence, they are very attractive for wearable health monitors, environmental sensors, and in energy systems. Moreover, self-powered electronics is gaining attention recently, leading to increased research on these areas [11]. Apart from wearable devices, these self-powered energy devices can also be efficiently used for vehicle transportation [12]. As these portable, self-powered, and renewable devices provide a sustainable energy source avoiding separate batteries and charging units, they are very important for sustainable development of economic and human lifestyle in the future. However, the use of wearable devices is limited due to high cost, challenging scalability. Therefore, cost-effective materials and methods are highly expected to produce these sustainable energy devices. In this review, the different techniques available to convert environmental energies without exploiting fossil fuels are consolidated, focusing on ZnO and its advantages. The contents of this review report are consolidated in Table 1 for an overview of the manuscript.

## 2. Oxide Materials for Wearable Nanogenerators

Generally, a variety of inorganic and organic materials are used for the fabrication of wearable nanogenerators. These materials can vary depending upon the kind of nanogenerators, such as piezoelectric generators, triboelectric generators, and others [13]. However, the oxide materials display a wide range of properties that facilitate their use in many product areas; these materials help manufacturers of thin-film PV cells achieve greater efficiency [14]. The unique physical, thermal, and electrical properties of oxide materials make them reliable, highly durable, and cost-effective for use in harsh environments, often found in solar cell and other energy device manufacturing. As they feature high hardness, physical stability, extreme heat resistance, and chemical inertness, oxide materials are used to prepare enclosures for solar cells and other devices needed solid covers.

## 3. Advantages of ZnO as a Best Alternative Material for Hybrid Energy Harvesting Applications

Recently, considerable attention has been focused on oxide materials for high-technology applications that depend critically on the ability to tailor structures and properties to optimize the interplay between form and function. Oxide materials are also a forefront area in materials science, which commonly includes oxides, nitrides, and carbides. There are numerous metal oxides, such as ZnO, CuO, TiO_2_, SnO_2_, NiO, CoO, Co_3_O_4_, etc., that are useful for varieties of applications [15,16]. Among these, the thin films of TiO_2_ and ZnO have attracted attention for energy conversion applications [17,18,19]. ZnO is a multifunctional material that can be used for a variety of applications through modification of its surficial and compositional properties. It can be used for photovoltaics, photocatalytic, bio and chemical sensors, field effect transistors, piezoelectric and thermoelectric applications [19,20,21,22]. ZnO is highly attractive for variety of applications, it is a II-IV n-type semiconducting material with piezoelectric and thermoelectric properties [23]. Moreover, nanodimensional rod and sheet shaped ZnO structures can easily be prepared using the low-cost hydrothermal method for a variety of applications including nanogenerators [6].

### Properties of ZnO Thin Films

**Figure 3:**
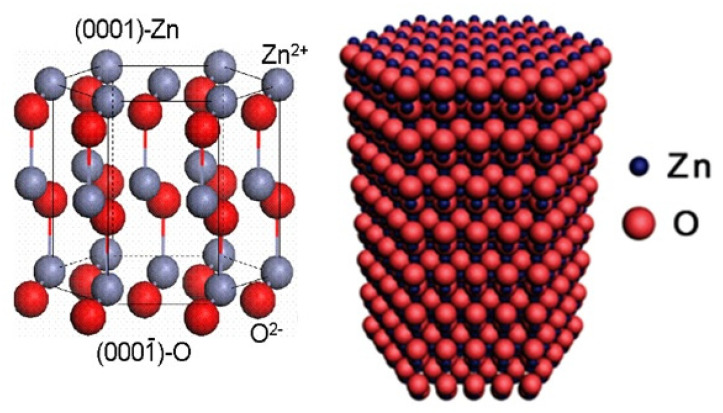
Crystal structure of wurtzite ZnO.

ZnO is a unique material with both semiconductor and piezoelectric properties and can be synthesized by inexpensive low-temperature techniques [21,24]. ZnO is a wide bandgap (3.37 eV) semiconducting material [20] that can exhibit UV luminescence and excitonic emission at room temperature. ZnO has high transparency in the visible region, and its conductivity can be improved by doping with different metals. It is a wurtzite crystal structured material lacking in center of symmetry with large electromechanical coupling, this makes it both a pyroelectric and piezoelectric material. This hexagonal ZnO is composed of O^2−^ and Zn^2+^ ions stacked alternatively along the c-axis, as shown in Figure 3. It has a direct bandgap of 3.37 eV with large exciton binding energy of 60 meV at room temperature. It is found in the zincite mineral as white powder soluble both in water and alcohol. It has a hexagonal wurtzite structure with lattice parameters a = 3.249 and c = 5.206 Å, and space group p63mc. The n-type behavior of ZnO is produced by the presence of oxygen vacancies with excess zinc atoms in interstitial positions. It also has excellent chemical and physical properties showing high electrical mobility with low toxicity. In addition, it can be synthesized easily at low-temperature with low-cost. As it can be grown at low-temperature, it is suitable for growth on polymer and flexible substrates. Moreover, it has good mechanical and chemical stability suitable for high temperature and high voltage applications. This high chemical, thermal, and mechanical stability, along with the biocompatible nature of ZnO, makes it attractive for wearable and implantable devices [25]. Even though ZnO is a promising material for thermoelectric applications, its high thermal conductivity and poor electrical conductivity limits the application. However, the strategies of nanostructuring and doping improve the properties of ZnO for piezoelectric and thermoelectric applications.

## 4. ZnO for Solar Energy Conversion

Given the fast growth of PV technologies, there is a race to improve the performance of PV cells to reduce the total cost of electricity generated to be cheaper than those from other sources [26]. The basic building block of a PV system is the thin film solar cell. This solar cell is a semiconducting solid-state device used to convert solar light into electricity. In a typical solar panel fabrication, several units of individual solar cells are connected to produce solar panels or modules. To give structural integrity, all the individual solar cells are fixed on atop rigid flat surfaces or substrates. To provide electrical insulation and protection against environmental corrosion, solar cells are often enclosed using transparent and rigid materials [27]. For the purpose of the encapsulant and support structures, oxide materials are widely used [28].

ZnO and other important materials have been used for various applications, including semiconductor and optical components, decorative and low-emission architectural glasses, and most recently in the manufacture of flat screens for TVs and computers [29,30]. In solar cell production, thin films of solar active materials such as copper indium diselenide, cadmium telluride, and copper indium gallium diselenide alloys offer simpler and inexpensive alternatives to costlier crystalline silicon wafers [31,32]. Most of the efforts in this direction are now centered on thin-film deposition rather than wafer-based modules as these incur large costs. The favorable factors of thin films are that they use less materials and are much faster and simpler to manufacture than complex and delicate wafer processing techniques. This means that if the cost of deposition is reduced, then the efficiency of the resulting PV cells can be increased sufficiently. The conventional monoband gap solar cells (p-n junction) cannot utilize the entire UV to IR wavelength range of the solar spectrum; hence, a majority of the solar radiation is wasted and efficiency is reduced [33,34]. Thus, there is great interest in developing solar cells that can convert the entire spectrum of solar light in recent times. Therefore, considering the advantages of oxide materials, particularly ZnO, it has attracted enormous attention in the fabrication of solar cells including dye-sensitized solar cells, which can reduce the processing costs to reasonable levels and thereby increase production efficiency.

### 4.1. Dye-Sensitized Solar Cells

Before the invention of dye-sensitized solar cells (DSSCs) in 1991 by Michael Grätzel and Brian O’Regan, solar cells were dominantly fabricated as inorganic solid-state junction solar cells. DSSCs have attracted abundant attention due to their high efficiency, low-cost preparation method, and materials [35,36], which have rendered them as active contenders to conventional silicon-based solar cells. These photoelectrochemical DSSCs are used by photosensitization using wide-bandgap semiconducting mesoporous oxide materials. The DSSCs and conventional inorganic solid-state solar cells are totally different in terms of functional components. In DSSCs, the photoelectrons are generated by the semiconductor film and dye molecules that are separated from the charge carriers. The major advantage of dye sensitization is the majority carrier-based conduction, as compared to the minority carrier-based transport in conventional inorganic cells. This means that bulk or surface recombination of the charge carriers in the semiconductor are prevented. Thus, low-cost materials with inexpensive simple processing methods are permitted. Hence, this allows development of promising low-cost devices for photoelectrochemical solar energy conversion.

### 4.2. Operational Principles of DSSCs

DSSCs are different from conventional solar cells regarding the mechanism of light-to-electricity conversion [37]. In conventional solar cells, the semiconducting silicon p-n junction provides photoelectrons, whereas in DSSCs, photoelectrons are generated by the dye, and the semiconductor separates the charge in association with an electrolyte solution using photoelectrochemical regenerative process [38,39]. The main difference of this DSSC compared to conventional solar cells is the functional element for light absorption (i.e., the dye). The main components of the DSSC include (1) a transparent conductive fluorine-doped tin oxide (FTO or SnO_2_:F) substrate as the photoanode and a mesoporous film (10–15 μm) of a wide-bandgap semiconductor like TiO_2_, SnO_2_, and ZnO nanoparticles, (2) a monolayer of organic dye molecules (sensitizer) adsorbed onto the semiconductor nanoparticles, (3) a layer of electrolyte containing iodide/triiodide (I_3_^−^/I^−^) redox couple, and (4) a platinum coated FTO counter electrode. The electrolyte solution is filled between the photoanode and counter electrode to form the sandwiched solar cells. FTO: F-SnO_2_ coating is used as a TCO material due to its stability at high temperatures and has reasonable conductivity.

**Figure 4:**
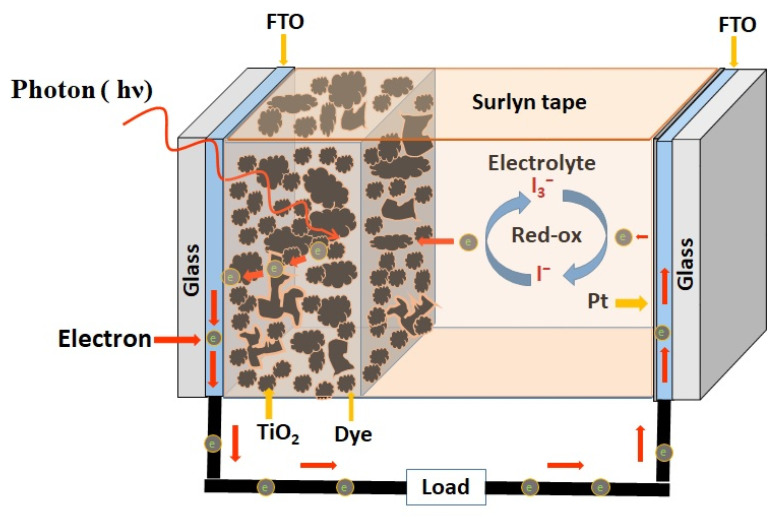
Schematic structure and mechanism of a dye-sensitized solar cell (reproduced from [40]).

The structure and operating mechanism of a DSSC is shown in Figure 4 [40]. Under irradiation by sunlight (photons), the dye molecules adsorbed on the nanocrystalline wide-bandgap semiconducting particles are excited, which inject electrons into the conduction band of electrode material; these injected electrons migrate to the front electrode (photoanode) and can be extracted as external current. Then, the oxidation reaction produced in the electrolyte restores the original state of the dye by electron donation from the oxidation reaction of iodide, and the electrolyte is regenerated by the reduction reaction of triiodide at the counter electrode. Thus, the redox electrolyte (iodide/triiodide) makes the process continuous. The electrons generated at the dye–semiconductor interfaces are conducted toward the counter electrode via an external load completing the circuit. The Pt-coated counter electrode acts as a catalyst for the redox reaction and for low-resistance electron transfer. As there is no permanent chemical change during this generation of electric power, this device can be used to continuously extract power from the sun [41].

The main issues of DSSC are its stability against time duration and temperature variation. Impure materials can also reduce the lifetime of the cells, and the behaviors of the liquid electrolyte can also change under extreme conditions. Therefore, for successful fabrication of DSSC for commercialization purpose, encapsulation and sealing are imperative, where oxide materials play strong roles [42].

### 4.3. Advantages of DSSCs

The fundamental advantages of the DSSC are efficient charge collection and suppression of charge recombination [43,44]. The fabrication of DSSCs requires simple processing techniques without sophisticated clean room facilities. Low fabrication cost and compatibility with flexible substrates are some of the other benefits of this device. The efficiency of DSSCs can be improved further by modifying the dye materials and electrolytes for efficient hole-transporting. To make them commercially feasible and economically viable technologies, DSSCs should have stable operational performance without degradation for several years. ZnO shows poor responses in DSSC performance owing to its instability in the acidic dye. Instead of using only ZnO, its combination with other materials or functional-material-sensitized nanostructured ZnO can improve DSSC performance. In this case, combination of ZnO with TiO_2_ can boost its performance [44]. The problem of long-term stability of the DSSCs can be improved by modifying the TiO_2_ film surface and slowing the photochemical degradation of the dye. Porous nanocrystalline TiO_2_ and ZnO films are usually employed as the photoanodes of DSSCs for absorbing dye molecules, which play important roles in the light harvesting efficiency of DSSCs. Quantum dots like CdSe can also be incorporated with the nanocrystalline thin films to enhance the photoconversion efficiency [45].

Semiconductor oxides like TiO_2_, ZnO, SnO_2_, and Nb_2_O_5_ are used in the fabrication of DSSCs. Owing to its nontoxicity, biocompatibility, and low-cost, TiO_2_ [46] is the best choice among other semiconductors. Gratzel first used TiO_2_ nanocrystalline films with ruthenium dyes and iodide/triiodide to fabricate DSSCs and convert solar radiation into electrical energy. Nanocrystalline TiO_2_ film is first coated on a transparent conducting substrate; to spread the viscous dispersion of colloidal TiO_2_ particles on the transparent conducting substrate, the doctor-blade and screen-printing methods are widely used. Nanocrystalline TiO_2_ films provide large surface-to-volume ratios, which are useful for more adsorption of the dye molecules. TiO_2_ DSSCs have been reported to provide solar to electricity conversion efficiencies greater than 10% under AM 1.5 irradiation. Hence, similar to TiO_2_, ZnO is also attractive for DSSCs.

### 4.4. ZnO-Based DSSCs

Several combinations of semiconducting materials and dyes have been studied to improve the efficiency of DSSCs and make this technology commercially viable. The DSSC using TiO_2_ and RuN_3_ dye has reported the highest efficiency of 11.2%. Although TiO_2_ film has achieved the highest efficiency out of all semiconductors, it has a significant limitation when growing morphologically controlled structures using a wide variety of substrates. One-dimensional (1D) structures of metal oxides, such as nanorods (NRs) and nanowires (NWs) will greatly improve the efficiency of DSSCs. Various oxides such as ZnO, In_2_O_3_, Nb_2_O_5_, and SnO_2_ have been studied extensively to improve DSSC performances. Among these materials, ZnO has been identified as a suitable material for TiO_2_ because of its higher electron mobility, similar bandgap as TiO_2_, and potential in producing a variety of morphologies [47,48].

### 4.5. One-Dimensional ZnO NW Films

**Figure 5:**
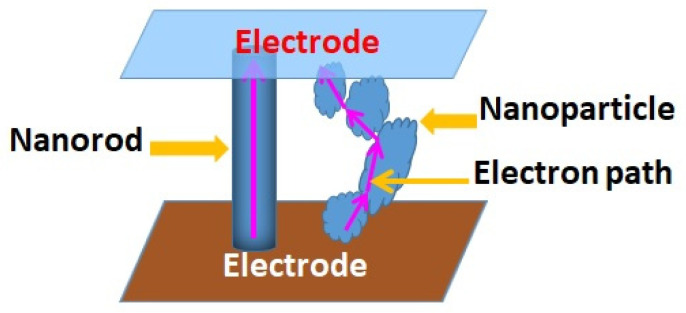
Electron path difference between nanorods and nanoparticles.

In the case of DSSCs, the dye molecules directly attached to the semiconductor surface are only able to liberate charge carriers efficiently. Films of semiconducting nanoparticles offer limited surfaces for the adsorption of the dye monolayer causing reduced efficiency [49]. This can be avoided by using nanoporous semiconducting materials as they provide more area for dye adsorption [50]. The design of a DSSC should be such that the optical losses are minimized, and light-collecting efficiency of the device is improved. Moreover, it should ensure the efficient transport of charge carriers to get current. The morphology of the semiconducting material used should be highly porous, so that it can provide high surface area for maximum dye adsorption with efficient charge carrier transport to deliver the photoelectrons to the collection electrode without recombination. Photoanodes made up of 1D nanostructures can be effective for facilitating electron transport and improving efficiency. Compared with nanocrystalline particle films, films containing vertically aligned ZnO NRs favor electron transport owing to the easy electron transport without any interaction. Law et al. found that the electron diffusion coefficient of ZnO NWs is higher than the films of TiO_2_ and ZnO nanoparticles [51]. Replacement of nanoparticles with NWs may be advantageous because the NW provides direct and smooth conduction paths for the electrons while providing high surface area for dye adsorption, as shown in Figure 5. The barrier induced by the nanoparticle boundaries can reduce conduction.

**Figure 6:**
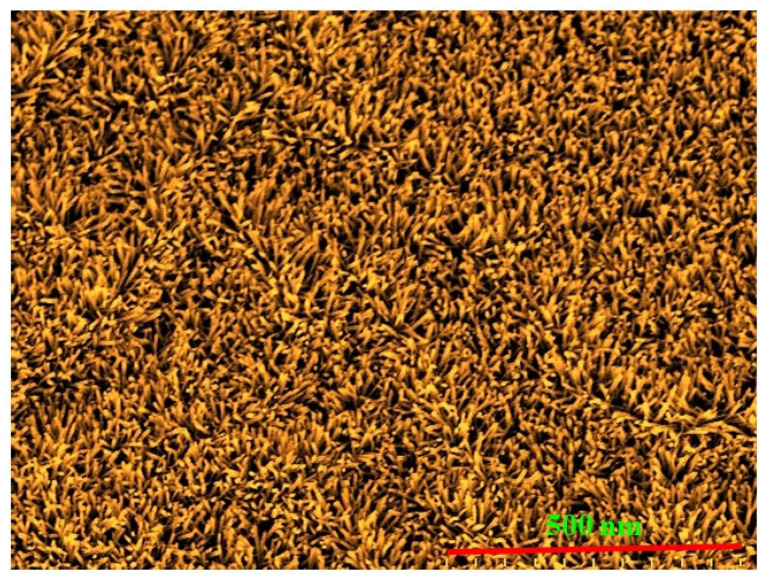
ZnO nanowire film grown via simple solution method at low-temperature.

ZnO nanostructures such as 1D NW, NR, nanoring, nanohelix, and two-dimensional (2D) nanosheet/nanoplate have recently attracted attention considering their advantages [52]. Among these, the highly ordered 1D ZnO nanostructures are highly important to develop novel energy devices, such as PV cells, as well as nanogenerators and sensors. The main advantage of one-dimensional ZnO NRs is the reduction in carrier path length; the carrier transport is made easy and efficient due to the straight-line baths in the nanorods. The vertically aligned NRs directly link the electrodes without any lateral contact producing smooth electronic transport with high mobility [53]. The electronic transport in highly ordered crystalline nanorod arrays is very high compared to randomly oriented polycrystalline network of NRs. Dense array of long and thin NRs is possible to increase dye loading by maintaining very good carrier collection in DSSC. This high transport provided by NRs is particularly very useful to design cells using polymer gels electrolytes, in which the recombination rates are high compared to those of liquid electrolyte cells [54]. High-quality vertical ZnO NW arrays are grown using the gas-phase epitaxial method; however, reasonably aligned ZnO nanowires can be grown on any kind of substrate using low-temperature solution methods (Figure 6) [55], making it an attractive material for energy conversion devices.

### 4.6. Nanowire and Nanoparticle Composite Structure

**Figure 7:**
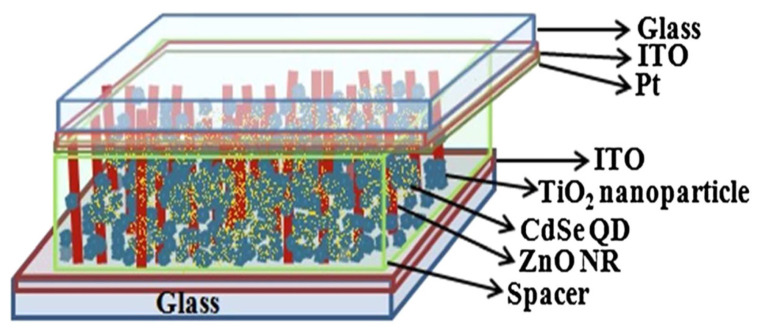
Structure of DSSC incorporating TiO_2_ nanoparticles and CdSe quantum dots with ZnO nanowire film (reproduced from [40]).

Photoanodes made up of one-dimensional nanostructures like ZnO NRs or TiO_2_ nanotubes show low surface areas compared with porous nanoparticles (NPs) used in photoanodes, which result in decreased photo-to-electric efficiency owing to the low adsorption of dye molecules. Therefore, photoanodes with one-dimensional NRs or nanotubes mixed with NPs, as shown in Figure 7, are used to effectively improve the overall cell performance [40].

Although there are various improvements to increase efficiency since the invention of DSSCs, it still suffers recombination problems of the charges injected into the electrolyte. Qin Hu et al. proposed the idea of using core/shell structures for photoanodes to suppress recombination and reduce electron losses [56]. In such structures, a wide-bandgap semiconductor SrO (Eg = 5.7 eV) coating is applied on the surface of the porous one-dimensional NW and NP film to form a composite core–shell structure of the photoanode. In this photoanode structure, NWs enhance electron transfer and light scattering effects, and the NPs included increase the surface area useful for efficient and more dye adsorption, the SrO shell coating provided suppressed recombination of the charges in the electrolyte. Therefore, the photo-to-electric conversion and the charge transfer can be improved in this novel photoanode.

## 5. ZnO Nanowire Piezoelectric Nanogenerator

Recently, several advances have been reported in electronic industries, including artificial intelligence (AI)-dominated robotic facilities. Completely self-powered electronic instruments are highly needed for this automatic world, which necessitates miniature energy harvesting devices. In this case, nanogenerators that are able to convert the background environment energy are important in the AI-dominated robotic world. Harnessing the environmental energy and directly converting different forms of environmental energies into useful forms are some of the attractive and innovative approaches garnering attention from researchers [57]. This is also a promising approach for powering nanodevices. Wireless and implanted nanodevices demand self-powered and maintenance-free nanogenerators to work sustainably without requiring a battery or recharging processes [58]. ZnO nanowire (NW) devices have attracted immense interest as nanogenerators to convert mechanical vibrations (sound) and light simultaneously using their unique semiconducting, piezoelectric, and photoelectric properties [59]. Recently, considerable efforts have been invested in the development of such nanogenerators by coupling the piezoelectric and semiconducting properties of ZnO NWs [60].

Mechanical energy in the form of sound waves, mechanical vibrations, air flow etc. is available abundantly in the environment [61]. These mechanical vibrations are converted into electrical energy using the piezoelectric effect of ZnO NWs. ZnO NWs are unique for the fabrication of nanogenerators to scavenge the mechanical energy due to its piezoelectric nature [62]. This mechanical energy conversion into electrical energy from ambient sources is very useful to power electrical devices without need for separate batteries. Bodily movements such as walking, breathing, and other muscle movements can also produce strain on the ZnO NW, which could be used to produce self-powered wireless and implantable nanodevices like pacemakers. One creative initiative is to use ZnO NWs to prepare self-driven nanodevices using only ambient energies without requiring external power sources [63].

### 5.1. Concept of ZnO Nanowire Piezoelectric Nanogenerator

The concept ZnO NWs-based piezoelectric nanogenerator was reported first by using atomic force microscope [64]. The lack of centerosymmetry in the crystalline ZnO NR structure results in piezoelectric effect, and hence, the mechanical stress/strain changes are converted into electrical signal utilizing this effect and vice versa, owing to the relative displacements of the cations and anions in the crystal [65]. Breaking the central symmetry of wurtzite structured ZnO crystal by external force creates a piezoelectric potential. In an undisturbed state, the charge center of anions (O^2−^) and cations (Zn^2+^) coincide symmetrically along c-axis. When an external force is applied, the charges are separated, forming an electric dipole and creating a piezopotential due to piezoelectric effect, which causes a current flow in the external circuit.

**Figure 8:**
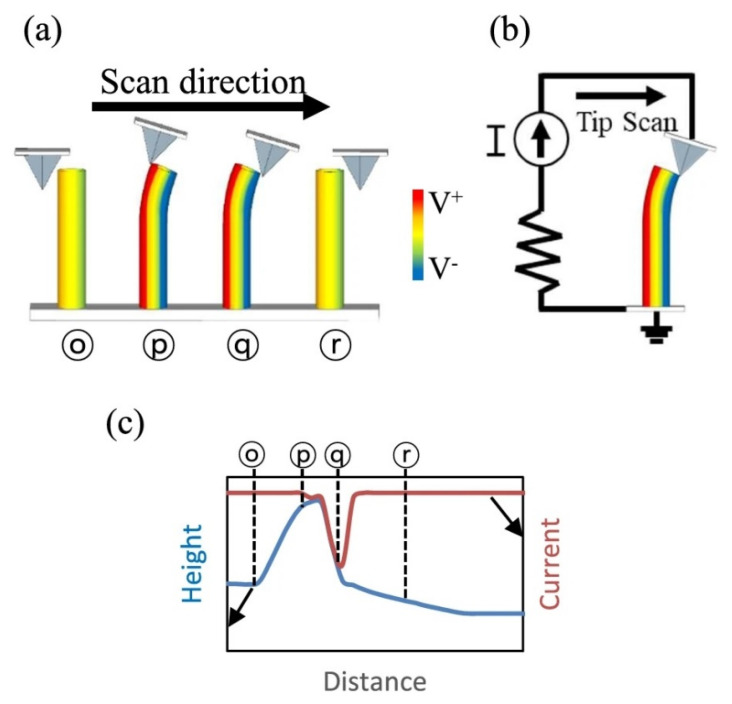
(**a**) AFM tip movement on ZnO nanorod; (**b**) Piezoelectric generation; (**c**) AFM tip position dependent output signal (reproduced from [66]).

The power output mechanism of a nanogenerator is achieved by the coupling effect of piezo and semiconducting properties of ZnO. Wang et al. confirmed this effect by scanning the surface of a ZnO NW film using contact-mode AFM by applying a force between the tip and electrode surface [64]. The piezopotential was measured across an outside load. When the tip was scanned on the vertical NWs, the NWs were bent successively, and the corresponding potential was recorded (Figure 8) [66].

Thus, the current recorded is due to the piezogenerated signals (Figure 8c), caused by the bending of the NW as the AFM tip moves over the ZnO NW film. This effect was observed only in piezoelectric nanowires; when tested using NWs of other nonpiezoelectric materials like tungsten oxide, no electrical signal was observed when the wires were bent. The signal produced is sinusoidal depending on the bending direction; to draw unipolar signals, the contacts should be in such a way as to rectify the signal. The generated signal is rectified due to the Schottky diode formed between the contact metal and ZnO NW; this is the important feature for current generation and the output process of the nanogenerator [67]. The mechanism of charge generation and separation can be explained according to piezoelectric effect of ZnO NW. In a vertical NW, the bending of the NW by application of a force produces a strain, with compression in inner surface and stretching in outer surface. As a result, a piezoelectric potential is built up in the NW with positive in stretched side and negative in compressed side. The displacement of O^2−^ and Zn^2+^ of ZnO due to the piezoelectric nature creates a potential. These separated ionic charges are stable maintaining the formed potential difference until applying the releasing force. When a proper contact is made, there is a flow of charge constituting a current flow. This ZnO-NW-based nanogenerator can effectively be used to harvest environmental mechanical energy to power implanted and remote nanolevel devices. It is a breakthrough invention for the development of self-powered devices by harvesting environmental energies using piezoelectric nanogenerators.

### 5.2. Horizontally Aligned Nanowires for Piezoelectric Nanogenerator

The voltage produced by a single NW is insufficient for real devices. Hence, we need to integrate large numbers of NWs into a single power source. A single NW aligned parallel to a flexible substrate can be deformed or agitated by environmental vibrations to piezoelectric generation [68]. This current level may be very less; hence, to enhance the output power, multiple NWs should be integrated [69]. When the outputs of many NWs are integrated, several factors should be considered. There should be at least one Schottky contact to rectify the signal, and all the contacts should be robust enough to withstand mechanical deformations. To obtain enhanced output voltages and current improvements in the interconnection of electrodes and NWs, the strain or straining rate is important. Both horizontally and vertically aligned NWs can be used on either rigid or flexible substrates depending on the stain application method. Guang Zhu et al. fabricated flexible high-output nanogenerators using horizontally aligned arrays of ZnO NWs producing 2.03 V (Voc) and ~11 mW/cm^3^ power density [70]. Sheng Xu et al. grew ZnO NWs aligned parallel to the substrate between gold contacts, using a chemical approach [62]. The structure was deformed using a periodic external force to subject the NWs to a cyclic stretching–releasing deformation process. When all the NWs on a substrate are subjected to bending and releasing forces, there is charging and discharging that causes an alternating signal generation. If the charging and discharging processes of many NWs are synchronized properly, a high a.c. output voltage can be generated.

### 5.3. Vertically Aligned Nanowires for Piezoelectric Nanogenerator

**Figure 9:**
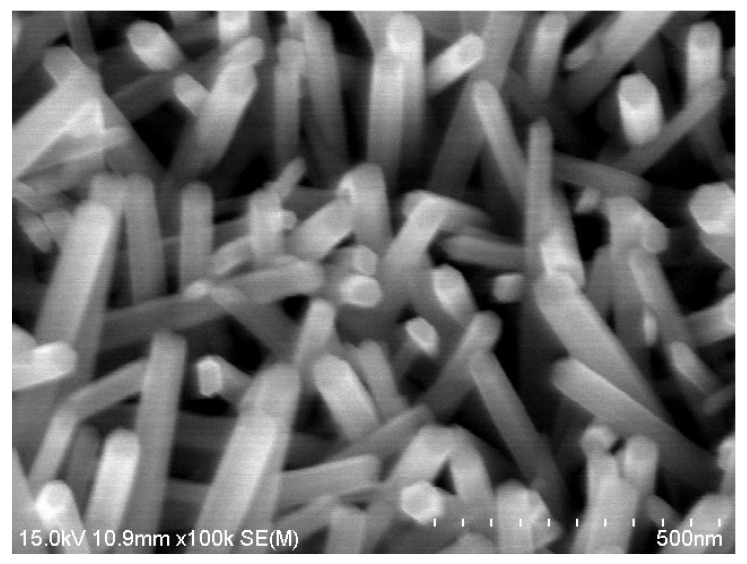
SEM image of the solution-synthesized ZnO nanowire film.

Vertically aligned NWs and NRs are extremely attractive compared to horizontally aligned ZnO NWs owing to their simple fabrication processes. Kathalingam et al. reported ZnO NWs of length ~1 µm and diameter ~50 nm on p-Si substrate (Figure 9) [55]. These ZnO nanowires were grown vertically on the substrate using the hydrothermal route after forming a seed layer by spin coating on p-Si. Platinum metal contacts were coated on the tips of the NWs and as contact pads on the p-Si substrate. The platinum coating on the tips of the NRs was achieved by oblique incidence using an E-beam evaporator.

**Figure 10:**
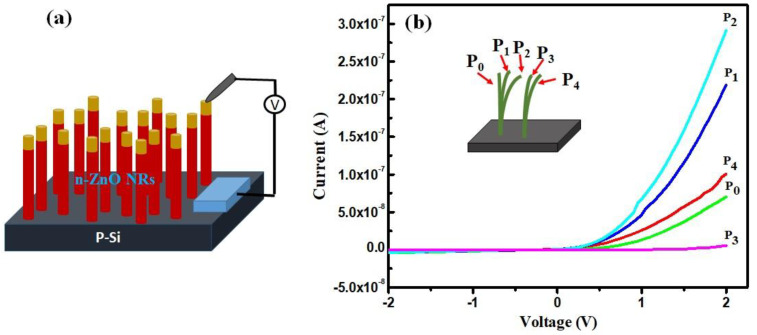
(**a**) Schematic illustration of *I*–*V* measurement; (**b**) *I*–*V* curve of the device for different applied pressure values [55].

The platinum metal forms Ohmic contacts with the ZnO NW, and the p-type silicon forms a p-n junction with the n-type ZnO. Hence, the piezogenerated signal can be rectified in this design. A periodic bending force was applied tangentially to the tip of the ZnO NWs, as shown in Figure 8a, and the current was recorded. Figure 10b presents the *I*–*V* curves recorded for every 5° rotation of the knob of the measurement tip. The contact tip positions on different ZnO NWs for increased motion are denoted by P_0_–P_4_, as shown in the inset of the Figure 10b. When the application pressure is increased from P_0_ to P_4_, the current increases (P_0_, P_1_, and P_2_) first and then decreases to P_3_ before increasing again at P_4_. This application of the pressure bends the NWs and results in piezoelectric generation, which is attributed to the increase in current with applied pressure. The decrease of current for P_3_ position is due to the contact of the probe with the next NW, whose further bending again increases the current at P_4_.

**Figure 11:**
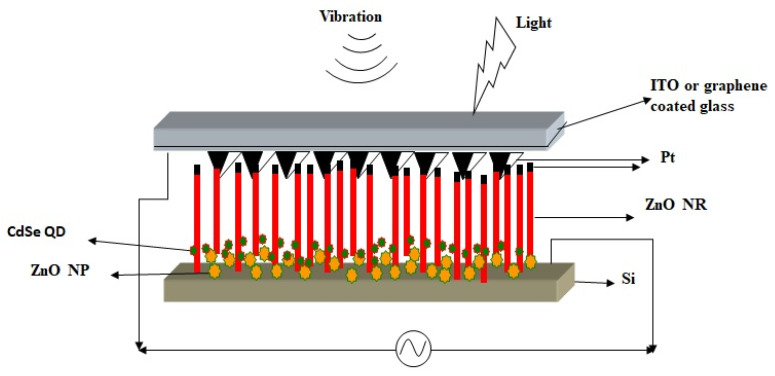
Piezo- and photo-coupled nanogenerator.

The energy generated by all the NWs should effectively be collected simultaneously and continuously before being converted as useful output. Instead of using a single probe like the AFM tip, new probes and innovative approaches have to be developed. A common conductive electrode can be used to simultaneously collect the generated signal; it is more advantageous if it has a trench-shaped metal coating, as shown in Figure 11.

**Figure 12:**
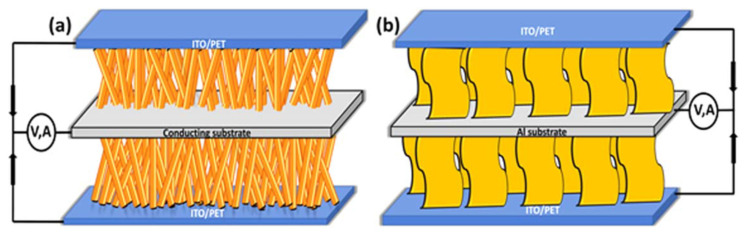
Device design with (**a**) nanorods array, and (**b**) nanosheets array (reproduced from ref. [71]).

When the top electrode moves vertically or back and forth depending on the background vibration or due to any other oscillation, the NWs bend and generate a piezoelectric potential. The trench pattern ensures simultaneous contact with all the NWs and further conduction. Vertically grown ZnO NWs have the advantage of being integratable with ZnO NPs and other photosensitive NPs, so that the structure can be used to harvest both light and sound (mechanical vibrations) simultaneously. The substrate p-Si makes a p-n junction with the ZnO NPs to form a solar cell, and this structure can also convert incident light if the top electrode is transparent. Mishra et al. has produced ∼285 mV from a ZnO nanosheets based PENG, devices, in which they have grown ZnO nanosheets and nanorods array on double sides of aluminum sheets using hydrothermal technique, as shown in Figure 12 [71]. Thus, ZnO is the best oxide material for growing well aligned nanorod arrays.

## 6. Piezoelectric- and Photoelectric-Effect-Coupled Nanogenerator

**Figure 13:**
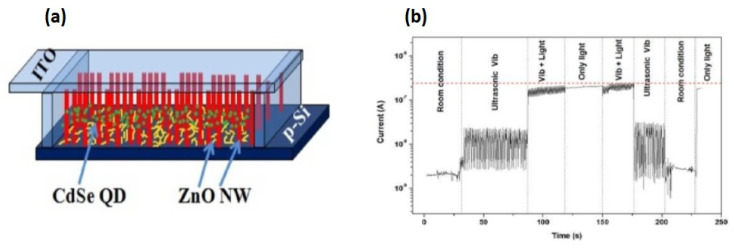
(**a**) Schematic of the piezoelectric and photoelectric integrated nanogenerator and (**b**) electrical current produced from vibration and light (reproduced from [59]).

Piezoelectric and solar cell hybrid nanogenerators can convert simultaneously sunlight and its ambient energies like sound, movements, etc. [72]. Fabrication of a piezo nanogenerator combined with photoelectric effect was demonstrated by Kathalingam et al. [59], in which ITO-coated glass plates were used as transparent conducting top electrodes (Figure 13a). They fabricated two types of devices, such as vertically aligned ZnO NWs alone as one and vertical NWs with horizontally deposited NWs as another. To incorporate free NPs with vertical NWs, a less-densely seeded film was used. An ITO conducting glass was used to cover the ZnO NWs grown on Si wafer as a sandwich-type cell. The thin layer of ZnO NPs formed over the p-Si in between the ZnO NRs forms a p-n junction solar cell causing photogeneration of carriers, with the ZnO NWs grown vertically on the p-Si acting as piezogenerators. This double-band structure of the ZnO/Si heterojunction efficiently collects solar light: high-energy photons in the ultraviolet region can be absorbed by the ZnO, while low-energy photons in the visible range are absorbed by p-Si after passing through the ZnO layer. The ZnO NWs grown along with c-axis provides strong piezoelectric effect due to the external force. The sound waves produced near the device vibrate the top contact electrode generating electric potential through the vertically well-aligned ZnO NWs. The piezoelectric and semiconducting (p-n junction) properties coupled with n-ZnO/p-Si structure could be used to fabricate nanogenerator capable of harvesting both solar energy and mechanical vibrations. The piezoelectric property of ZnO NW produces piezopotential due to mechanical bending, and the Si/ZnO p-n junction generates electric charges using photogeneration and associated rectifying nature. Hence, this hybrid system of the nanogenerator can simultaneously harvest both solar energy and mechanical vibrations, thus improving the power conversion efficiency (PCE).

This power generation mechanism relies on piezoelectric and semiconducting properties of the p-Si/n-ZnO heterojunction. Although this device converts simultaneously both mechanical and solar energies, their output signals are in AC and DC, respectively. To clearly observe the individual effects of the optical and mechanical energies on the device output signals, the current–time responses of the device were obtained with different sources. The device was tested for its responses to both light and mechanical vibrations using a halogen light (200 W) with ultrasonicator. First, the sonicator was switched on and a light was also incident on the device after a few minutes; then, the light was switched off leaving the mechanical vibrations alone for a few minutes before being brought to room condition. It was observed that the device condition was fully reproducible. This shows that the current induced depends on its background changes; application of ultrasonic vibrations has produced an AC-type signal, whereas irradiation of light has produced a pure DC signal with increased current level as expected (Figure 13b). For the application of both mechanical vibration and light, the overall output response of the device was synergistically enhanced.

**Figure 14:**
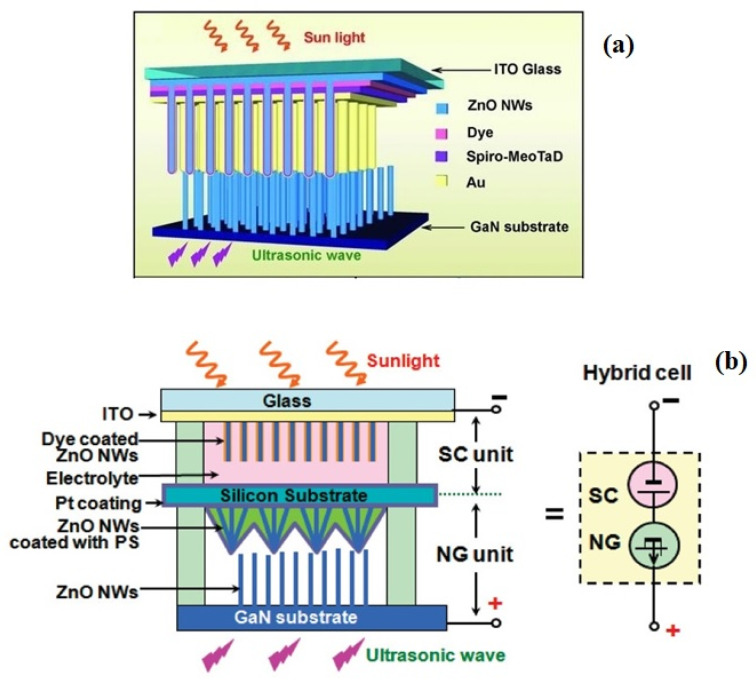
Schematics of solar cell and piezoelectric nanogenerator hybrid cells as (**a**) single cell and (**b**) sandwich type (reproduced from) [72,77].

This hybrid device can increase the overall current generation by the effect of combined solar and piezo electric generation of ZnO, so the resulting current could be the sum of the individual responses. The piezoelectric signal generated is rectified by the Schottky behavior of this p-n junction. This Schottky rectifying behavior separates the charges and builds the potential to generate electric current. This nanogenerator is capable of converting vibrations ranging from footsteps to ultrasonic sounds. The inclusion of CdSe quantum dots with the ZnO NW structure has also been found to increase the photoconversion efficiency [59,73]. ZnO nanoparticles (NPs) and its composites are also used for the fabrication of piezoelectric nanogenerators to convert ambient mechanical vibrations into electrical energy. Mahapatra et al. has fabricated flexible PENG by incorporating ZnO particles into PDMS matrix. They have further improved the performance by interfacing p-type NiO with n-type ZnO as heterojunctions formation [74]. Lee et al. has prepared ZnO based nanogenerator by inserting aluminum nitride insulating layers. This inclusion of insulating layer into the nanogenerator structure of ZnO and AlN stacked layers has created large potential barrier, useful for energy conversion [75]. ZnO-CuO p-n junction heterostructure based nanogenerator fabricated by Shin et al. has produced good performance in piezoelectric generation [76]. This p-n junction based heterostructure can also convert optical energy enabling the combined conversion of both mechanical and optical energy. A number of reports with different designs are available for ZnO NRs-based piezoelectric and photoelectric effects coupled nanogenerator to harvest simultaneously both solar and mechanical energies, as shown in Figure 14 [72,77].

## 7. Polymer-Incorporated ZnO Nanowires for Nanogenerators

**Figure 15:**
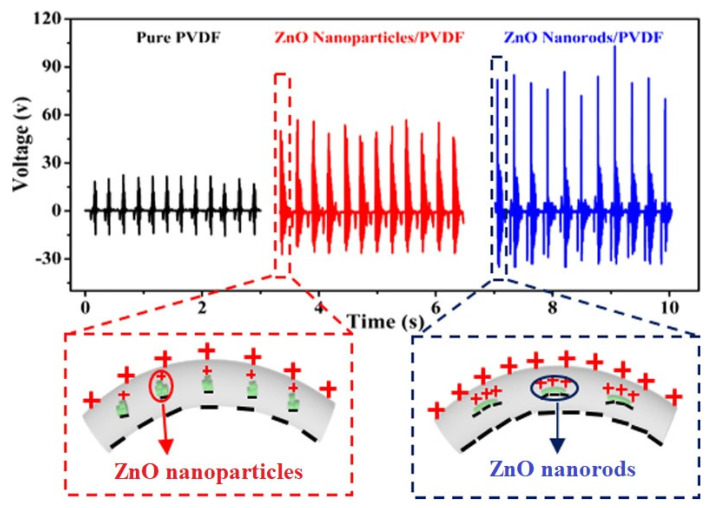
Difference of piezoelectric signal generated by pure PVDF, ZnO Nanoparticle/PVDF, and ZnO nanorods/PVDF (reproduced from [84]).

The conversion of mechanical and background energies into useful forms by ZnO nanogenerators offers several advantages, particularly for energy harvesting for small-scale and self-powered systems. Instead of using only ZnO NRs arrayed in films, the incorporation of a piezoelectric polymer with the ZnO NRs can produce efficient piezoelectric nanogenerators [78,79]. Incorporation of ZnO NWs with polymer is showing promising efficiency in converting mechanical energy into electrical energy. ZnO NWs and polymer composite structures, prepared vertically associating between two metallic electrodes, form an effective PENG device [80]. Several polymers can be incorporated with ZnO to fabricate hybrid energy harvesting nanogenerator devices. The incorporation of polymers with ZnO enhances the mechanical and electrical properties, particularly for piezoelectric energy harvesting. These different polymers include polyvinylidene fluoride (PVDF), polydimethylsiloxane (PDMS), polymethyl methacrylate (PMMA), polyurethane (PU), poly-L-lactic acid (PLLA), and PANI [81,82]. Among these, PVDF is one of the most used polymers owing to its highly flexible and piezoelectric nature. When used with ZnO, it forms an efficient nanogenerator to harvest mechanical energy due to its high piezoelectric and dielectric properties [83]. This polymer-incorporated piezoelectric nanogenerator can be modified to improve its piezoelectric properties, making it suitable for flexible wearable energy and sensor devices. Moreover, the inclusion of a polymer in the ZnO NR film helps to distribute the strain change uniformly throughout the structure. The added piezoelectric polymer can also enhance the contact area and durability of the ZnO NRs during mechanical strain. These polymer and ZnO combined nanogenerators scavenge environmental energies like light, sound, heat, and other mechanical energies using photovoltaic, piezoelectric, thermoelectric, and pyroelectric phenomena [11]. Normally, these nanogenerators convert wasted energy in the environment to useful forms for sustainability. This wearable form of nanogenerator can convert body movements, gas flow, sound vibrations, and any deformations produced into electrical energy. Compared to ZnO nanoparticles, nanorods-based polymer composite films are efficient for these piezoelectric nanogenerators. Li et al. has fabricated nanogenerators using ZnO nanoparticles and nanorods as fillers with PVDF matrix by electrospinning showing improved performance for nanorods used generators, as shown in Figure 15 [84]. It proves that polymer incorporated nanorod structures can produce good stability for mechanical strains during the piezo and tribo electric nanogenerators.

## 8. ZnO Nanostructures for Thermoelectric Nanogenerators

Among the various kinds of nanogenerators, the thermoelectric nanogenerator (TENG) has gained attention for the conversion of waste heat into electrical energy. There are a large number of thermoelectric materials that can directly convert heat to electricity. The high thermal conductivity and low carrier mobility of metal oxides result in smaller figures of merit (ZT), reducing the thermoelectric performance. Among the various oxides and other materials, ZnO has gained considerable attention owing to its improved thermoelectric performance, easy availability, low-cost, and nontoxicity. These thermoelectric generators are working based on Seebeck effect principle, where a thermocouple placed in a varied temperature transfers the charges causing an electric current. Moreover, flexible TENGs can be used as wearable devices to harness body heat, it is more beneficial for portable electronics. Normally, thermoelectric materials have limited efficiency converting only 15–20% heat into electrical energy. This also, surely will reduce the exploration of non-renewable sources of energy. There are different thermoelectric materials such as silicon germanium (Si-Ge), bismuth telluride (Bi_2_Te_3_), lead telluride (PbTe), SnTe. Though, they are interesting thermoelectric materials, their usage is limited due to poor chemical stability and durability. Moreover, they are toxic and require complex synthesis procedures. Whereas the metal oxides offer promising advantages compared to other materials in terms of stability. Most of the metal oxides have lower ZT values with low carrier mobility and high thermal conductivity. However, its ZT values are increased significantly at higher temperatures, which is entirely different from other thermoelectric materials [85]. Nanostructured and doped zinc oxides (ZnO) exhibit high ZT values.

The performance of ZnO’s thermoelectric conversion can be increased by doping or structuring as nanoforms like NPs, NRs, nanoribbons, and quantum dots. The nanostructured design decreases the thermal conductivity of ZnO, resulting in increased thermoelectric effect [85]. Al-Fartoos et al. developed thermoelectric glazing using aluminum-doped zinc oxide (AZO) and copper iodide (CuI) to harness waste heat from buildings for conversion to electricity. This has the potential to produce sustainably built environments while maintaining comfortable indoor temperatures [86]. Volkova et al. prepared a ZnO-based thermoelectric glazing nanogenerator by encapsulating ZnO NWs in polyvinyl alcohol (PVA), which showed improved electrical conductivity accompanied by increased Seebeck coefficient [23]. A novel miniaturized thermoelectric generator prototype preparation integrating p-type PEDOT:PSS and n-type Ga:ZnO has been reported by Lemine et al., which is useful for wearable devices [1].

## 9. Triboelectric Nanogenerators

**Figure 16:**
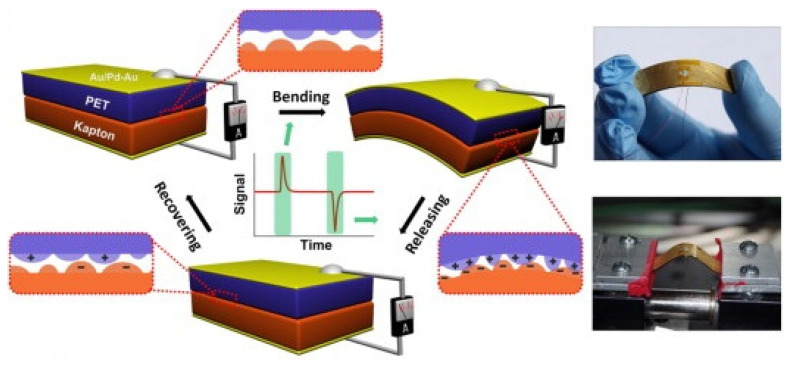
Schematic and working principle of polymer used triboelectric generator (reproduced from [88]).

**Figure 17:**
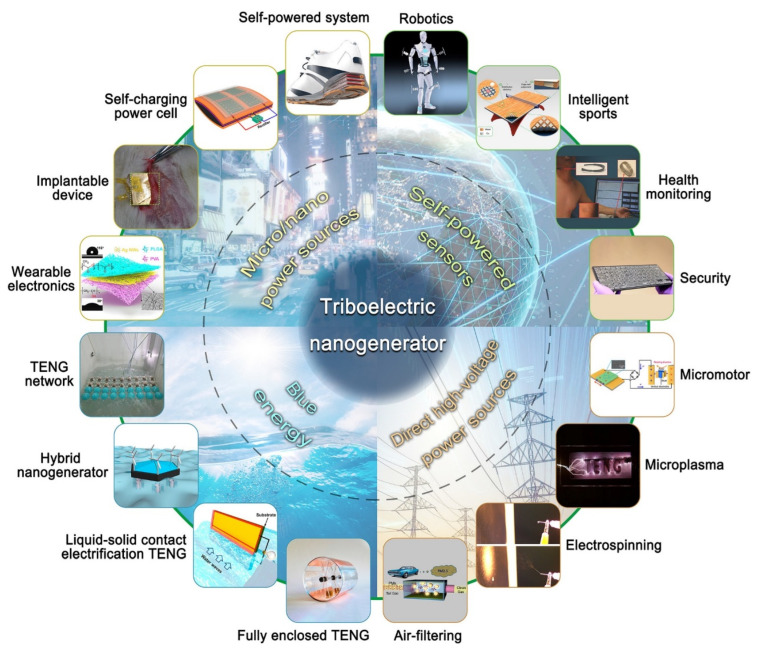
Different applications of triboelectric nanogenerators (reproduced from [90]).

When there is a relative motion of two materials surfaces via contact and separation, static electric induction is produced forming electric charges. This electric induction creates a potential difference causing a current in the external circuit. The triboelectric process-induced charges generation is usually considered as a negative effect, but the generated charges can be utilized for electronic devices, and further using these triboelectric generators wastes mechanical energies which can be converted into useful forms. ZnO-based composite films using MWCNT/ZnO/PDMS structure has produced good results for piezo and triboelectric effects coupled effect [87]. Fan et al. has demonstrated an efficient simple method to convert charges produced by friction into useful form for driving miniatured electronic devices [88]. The triboelectric generator was fabricated using stacked layers of different polymer materials with metal contact layers, as shown in Figure 16. When a mechanical strain is produced on the device, friction is produced between the layers causing a generation of opposite charges at the two layers. This triboelectrically induced potential drives the flow of electron, creating a current in external circuit. These triboelectric nanogenerators are produced from low-cost and compatible procedures, which have unique properties suitable for AI and robotic fields. The triboelectric devices can also be used for many intelligent systems due to their multidimensional sensing properties [89]. Moreover, this triboelectric nanogenerator (TENG) has a variety of applications, as reported by Luo et al. [90] (Figure 17). The triboelectric generator and solar cell hybrid nanogenerators can simultaneously respond to both sunlight and raindrops, producing electric current [91].

### Triboelectric and Piezoelectric Hybrid Nanogenerators

Triboelectric and piezoelectric hybrid nanogenerators can also be fabricated combining ZnO nanostructures with tribo-active materials in a hybrid design. These triboelectric and piezoelectric combined structures can effectively convert the backgrounds mechanical energies into useful form. The electrical energy obtained from these structures can usefully be employed for low signal applications. Efficiency of the tribo and piezo hybrid nanogenerators depends on the materials’ properties and design of the generators fabricated [92]. Mostly, ZnO or any inorganic nanoparticles matrixed with polymer materials are used for this combined nanogeneration fabrications. Diverse approaches can be adopted to combine both tribo and piezo materials for efficient harvesting of background mechanical energies. Inorganic nanoparticles are introduced into the matrix of polymer materials to enhance the triboelectric property of the composite structure. Among various inorganic materials, the ZnO nanoparticle is highly attractive considering its merits for these hybrid nanogenerators. Different nanostructures of ZnO such as nanoparticles, nanorods, nanosheets, and nanotubes are used for the production of tribo and piezo active structures. Recently, there are few works available on PDMS and ZnO nanowire arrays used nanogenerators for harvest energy from human body movements [93,94,95].

## 10. Light, Sound, and Thermal Hybrid Harvesting Nanogenerators

**Figure 18:**
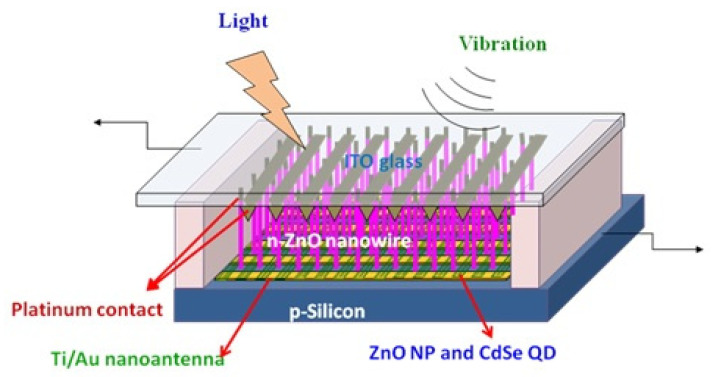
Light, sound, and thermal hybrid nanogenerators.

There is abundant energy in our environment in the form of light, sound, thermal, and mechanical energies. Harnessing these types of energies is critically important to fulfil the energy demands of mankind. Instead of harvesting these energies individually, combined harvesting of more than one form of energy offers more benefits in terms of energy and efficiency. This is also more useful for wireless and implanted nanodevices that demand self-powered and maintenance-free nanogenerators to work sustainably without requiring batteries or recharging processes. As in the environment, the human body also has bodily movements and temperature variations, which can be converted to power the implanted devices [96]. ZnO-NR-based devices have attracted immense interest in the field of nanogenerators as they convert mechanical vibrations (sound) and light owing to their unique semiconducting, piezoelectric, and photoelectric properties. Recently, numerous efforts have been reported in the development of such nanogenerators by coupling the piezoelectric and semiconducting properties of ZnO NRs. ZnO NRs with both piezoelectric and photoelectric properties can be synthesized easily using the simple solution route. ZnO is a promising thermoelectric material with a high figure of merit (ZT), high electrical conductivity, and Seebeck coefficient with high melting point. Hence, it can be used to harvest heat from the environment, vehicles, and power plants. However, its high thermal conductivity limits its usage, but alteration of its structure allows it to be used for heat conversion. Priyanka et al. modified ZnO by doping Al and showed improved thermoelectric properties [97]. Some oxide materials having ferroelectric property like BaTiO_3_ can extensively be used to harvest both optical and mechanical vibrations. Qian et al. has demonstrated flexo-photovoltaic coupled nanogenerator to harvest mechanical and optical energies using BaTiO_3_ based nanogenerator [98]. Pyrophototronic effect, a combined effect of semiconducting, pyroelectricity and optical excitation properties coupled nanogenerator fabricated using MXene/ZnO heterojunction has demonstrated as a light energy harvesting nanogenerator by Xue et al. [99]. By appropriate incorporation of infrared-sensitive nanoantenna structures in the ZnO nanostructures, along with vertically grown ZnO NRs on a patterned p-Si substrate, a nanogenerator could be fabricated, as shown in Figure 18, for simultaneously harvesting of light, sound, and heat; this will be a unique nanogenerator that can be used to power wireless and implanted devices.

The vertically formed ZnO NRs can convert background mechanical or sound vibrations, while the incorporated CdSe quantum dots and ZnO NPs can harvest optical energies. Simultaneously, the Ti/Au nanoantenna structures can convert infrared thermal radiation to useful electrical energy. Therefore, this hybrid structure can be used to harvest background energies for sustainable improvements in the future. Already, a number of reports are available on ZnO-based nanogenerators, and their responses are consolidated in the Table 2 for an overview.

## 11. Summary and Conclusions

In this review, recent progress of wearable flexible ambient energy conversion devices based on photoelectric, piezoelectric, and triboelectric, and their hybrid nanogenerators are summarized. The advantages of ZnO and its nanostructures for this hybrid energy harvesting applications are highlighted. ZnO nanostructures prepared as films or composite structures with other materials are attractive for various energy harvesting applications. The simple solution-prepared ZnO nanoparticles can be used efficiently for PV devices, including DSSCs to convert light to electrical energy. Quantum dots and other energetic materials incorporated in the ZnO NRs can form potential devices to harvest and sense optical energy. The inclusion of nanowire structures in application devices can enhance charge conduction owing to the inherent straight path, avoiding scattering-induced resistance. In nanoparticulate films, charges scattering and collisions are produced due to random walk of the charged particles at the interfaces. Moreover, the low-cost hydrothermal synthesis route of ZnO NRs facilitates low production cost and reduction of environmental damage by avoiding harmful chemicals. Composites of hierarchical ZnO nanostructures can be prepared using multiple solution methods and combined strategies, which can provide highly porous and high-surface-area films with efficient catalytic properties. ZnO NRs and conducting polymer composite films can be used to fabricate piezoelectric nanogenerators for harvesting background mechanical and sound vibrations. Conducting polymer coatings also offer the added benefits of uniform distribution of strain and durability increase to the ZnO NRs.

It is worth noting that in the future, ZnO-NR-based devices will be potential devices for a variety of advanced applications. Among the various piezoelectric materials, ZnO is a potential material for piezotronic and piezoelectric nanogenerators owing to its exceptional properties. This piezo- and semiconducting-coupled ZnO NR array offers multiple functions suitable for strain sensors, biosensors, gas sensors, humidity sensors, etc., along with piezotronic applications. This piezotronic effect can also be used in piezocatalysts, piezophototronics for photocatalysts, and photodetectors. More advanced technological devices will also be the focus in future industries based on ZnO-related materials. The involvement and integration of different functions of these materials are also expected to generate advanced technologies. Substantial progress has already been made anticipating more advanced highly efficient wearable nanogenerators in the near future.

## Figures and Tables

**Table 1:** Topic-wise contents of the report.

Serial No.	Topic
1	Introduction
2	Oxide materials for wearable nanogenerators
3	Advantages of ZnO as a best alternative material for hybrid energy harvesting applications
3.1	Properties of ZnO thin films
4	ZnO for solar energy conversion
4.1	Dye-sensitized solar cells
4.2	Operational principles of DSSCs
4.3	Advantages of DSSCs
4.4	ZnO-based DSSCs
4.5	One-dimensional ZnO NW films
4.6	Nanowire and nanoparticle composite structure
5	ZnO nanowire piezoelectric nanogenerator
5.1	Concept of ZnO nanowire piezoelectric nanogenerator
5.2	Horizontally aligned nanowires for piezoelectric nanogenerator
5.3	Vertically aligned nanowires for piezoelectric nanogenerator
6	Piezoelectric- and photoelectric-effect-coupled nanogenerator
7	Polymer-incorporated ZnO nanowires for nanogenerators
8	ZnO nanostructures for thermoelectric nanogenerators
9	Triboelectric nanogeneratores
9.1	Triboelectric and Piezoelectric hybrid nanogenerators
10	Light, sound, and thermal hybrid harvesting nanogenerators
11	Summary and Conclusions

**Table 2:** ZnO-based nanogenerator works available with their structural and conversion effectiveness.

S. No	Material and Structure	Device Structure and Type	Performance	Ref
**1**	ZnO nanosheets	ITO/PET/ZnO/Al/ZnO/PET/ITO piezoelectric generator	∼285 mV ∼1.7 times greater than single side growth	[71]
**2**	ZnO and AlN nanoparticles composite	Ag/AlN/ZnO/ITO/PEN piezoelectric generator	AlN insulating layer increased output 200 times more	[75]
**3**	Nd-doped ZnO	Nd-ZnO/PVDF/MWCNT Piezoelectric generator	75.8 V as Voc with 28.8 µA	[100]
**4**	Doped ZnO and polymer structure	PVDF/KNN/ZnO piezoelectric nanogenerator	25 V and 1.81 μA	[101]
**5**	ZnO/Polymer	ZnO/PVDF Piezoelectric generator	85 V of Voc at 2.2 µA	[84]
**6**	ZnO with different polymers	ZnO-PVDF Triboelectric nanogenerator	42 V of Voc, 62 µW/cm^2^	[102]
**7**	CuO-ZnO heterostructure	PET/CuO/ZnO/Au/Cu Piezoelectric generator	7.5 V with 4.5 µA/cm^2^	[76]
**8**	ZnO/BaTiO_3_ composite	ITO/PET/ZnO/BaTiO_3_/PET/ITO Piezo and pyroelectric generator	7.2 V, 2.0 µA	[103]
**9**	ZnO/PDMS	3D tubular structure Piezo and triboelectric combined generator	42.6 mW/m^2^	[104]
